# Association of *FPGS* genetic polymorphisms with primary retroperitoneal liposarcoma

**DOI:** 10.1038/srep09079

**Published:** 2015-03-13

**Authors:** Chengli Miao, Ding Liu, Feng Zhang, Youxin Wang, Yanbin Zhang, Junhui Yu, Zhanzhi Zhang, Gang Liu, Bing Li, Xing Liu, Chenghua Luo

**Affiliations:** 1 Retroperitoneal Tumors Center, Peking University International Hospital, Beijing, P.R. China; 2 Department of Surgical Oncology, Beijing Shijitan Hospital, Capital Medical University (The 9^th^ affiliated hospital of Peking University), Beijing, P.R. China; 3 Department of Neurology, The Third Hospital of Xiangya, Central South University, Changsha, Hunan, P.R. China; 4 National Engineering Laboratory for Druggable Gene and Protein Screening, Northeast Normal University, Changchun 130024, Jilin Province, P.R. China; 5 Chinese Academy of Sciences Key Laboratory of Genome Sciences and Information, Beijing Institute of Genomics, Chinese Academy of Sciences, Beijing, P.R. China; 6 Beijing Municipal Key Laboratory of Clinical Epidemiology, School of Public Health, Capital Medical University, Beijing 100069, China

## Abstract

Primary retroperitoneal liposarcoma is generally regarded as a genetic disorder. We have retrospectively genotyped 8 single nucleotide polymorphisms (SNPs) in 6 candidate genes (*MDM2, CDK4, CDC27, FPGS, IGFN1*, and *PRAMEF13*) in 138 patients and 131 healthy control subjects to evaluate the effects of genetic factors on individual susceptibility to primary retroperitoneal liposarcoma in Chinese population. Three SNPs (rs2870820, rs1695147, rs3730536) of *MDM2* showed significant differences in single-loci genotypes and allele frequencies between case and control groups (*p* < 0.05). The minor allele G of SNP rs10760502 in *FPGS* (folylpolyglutamate synthase) gene was significantly associated with increased risk for primary retroperitoneal liposarcoma, compared with major allele A. Our data suggest that *FPGS* variant in Chinese population may affect individual susceptibility to primary retroperitoneal liposarcoma.

Soft tissue sarcoma accounts for about 1% of adult solid tumors, with 15–20% located in the retroperitoneum^1^. Although retroperitoneal soft tissue sarcoma is rare, it causes critical morbidity and mortality. Soft tissue sarcomas are composed of more than 50 different histological subtypes, each with specific pathogenesis and clinical outcome^2^. Retroperiotoneal liposarcoma is a subtype of liposarcoma, a malignant tumor of mesenchymal origin that may arise in any fat-containing region of the body. Liposarcomas are the 2nd most common (annually 2.5 cases per million) of all soft-tissue sarcomas following malignant fibrous histiocytomas. Primary retroperitoneal liposarcoma accounts for about 45% of primary retroperitoneal neoplasms^3^. This tumor typically arises in persons 40–60 years of age, without any sex difference in incidence^4^. There are 5 histological subtypes: 1) well-differentiated: ~54%, low grade; including lipoma-like; inflammatory and sclerosing; 2) myxoid: ~31%, low to intermediate grade; 3) pleomarphic: high grade; 4) round cell: high grade and 5) dedifferentiated: high grade. The pathological type of primary retroperitoneal liposarcoma determines the therapeutic outcome and likelihood of metastasis. Highly differential liposarcoma is classified as Grade I according to the Federation National des Centers de LutteContre le Cancer (FNCLCC) classification, and simple mucin-like liposarcoma is classified as Grade II^5^,^6^. A ring chromosome is indicated in many primary retroperitoneal liposarcomas. Altered p53 pathway may play a pathogenic role in tumor progression of myxoid malignant fibrous histiocytoma-like liposarcoma, a dedifferentiated subtype^7^. Previous studies have focused on amplification of the chromosomal region 12q13–15^8^, and oncogenes *MDM2* and *CDK4*^9^. However, whether genetic variation in those genes affects primary retroperitoneal liposarcoma risk is unknown. To fill this gap in knowledge, we studied 138 patients and 131 healthy controls to evaluate possible associations of 8 single-nucleotide polymorphisms (SNPs) of 6 genes (*MDM2, CDK4, CDC27, FPGS, IGFN1*, and *PRAMEF13*) with primary retroperitoneal liposarcoma risk. Genetic susceptibility markers may be used to identify high-risk individuals for the early detection and prevention of primary retroperitoneal liposarcoma.

## Methods

### Study population

This study included 138 patients with primary retroperitoneal liposarcoma (experimental group) and 131 control subjects (control group) hospitalized at the Beijing Shijitan Hospital affiliated to Capital University between January 2009 and January2013. Each patient in experimental group had a pathologically confirmed diagnosis of primary retroperitoneal liposarcoma. All subjects were Chinese Han ethnicity. Recruitment of patients was not restricted with respect to age or gender. During the same period control subjects were recruited from hospitalized patients with different diseases (including various types of cancer other than soft tissue sarcoma) at Shijitan Hospital. Experimental group and control group was frequency-matched by age at the time of enrollment (±5 years) and gender. All study participants were residents of Beijing. The inclusion criteria for this study were available DNA sample and risk factor information. Written informed consent for an interview and a blood sample donation has been obtained from each participate. The study has been approved by the Institutional Review Board of Beijing Shijitan Hospital, Capital Medical University and been conducted in accordance with all ethical guidelines. The following information was obtained by personal interview: smoking, drinking.

### Blood sample collection and DNA extraction

Peripheral blood samples (5 mL) were collected from each subject at the time of enrollment, immediately snap-frozen in liquid nitrogen and stored at −80°C before DNA extraction. Portions of the tissues were treated in the same way as the blood while the residual tissues were paraffin-embedded for histopathological diagnosis according to WHO criteria. Genomic DNA from tumor tissues and peripheral lymphocytes was extracted using a QIAamp DNA Mini Kit (QIAGEN Inc.) and stored at 4°C for immediate use. DNA quality and purity were assessed by agarose gel electrophoresis, and optical absorbance was measured at A260/A280.

The genetic variants analyzed in this study included 8 SNPs within 6 genes (*MDM2, CDK4, CDC27, FPGS, IGFN1, and PRAMEF13*). The six candidate genes were selected from 1) Illumina TruSight Cancer panel, which includes >1400 SNPs of 400 tumor related genes; and 2) SNP500 tumor database, which is provided by National Institute of Health, USA (http://snp500cancer.nci.nih.gov/). These genes have been shown to correlate with the susceptibility to tumors of digestive tract. Candidate genes were chosen based on their important functions in tumorigenesis from the TruSeq Amplicon - Cancer Panel (TSACP) provided by Illumina Inc. All SNPs were selected from the National Center for Biotechnology Information SNP Database (http://hapmap.ncbi.nlm.nih.gov/cgi-perl/gbrowse/hapmap28_B36/), based on their potentially functional location and validation status. The SNPs in the entire coding region (synonymous or non-synonymous), together with the flank regions 2 kb upstream and 1 kb downstream of each gene, were at top priority. The minor allele frequency (MAF) of those SNPs was ≥10% in the HapMap-Han Chinese from Beijing (HCB) databank (www.hapmap.org). The genes, chromosome regions, nucleotide substitutions, functions, reference SNP identification numbers.

### SNP beadchip assay

Genotyping was performed using the Illumina SNP Golden Gate Assay (Illumina, Inc., San Diego, CA, USA) according to the manufacturer's specifications. Briefly, 250 ng of genomic DNA was amplified at 37°C for 20 hours, and then the amplified DNA was fragmented and precipitated. The dried pellet was resuspended and hybridized to the beadchip. The hybridized beadchips were incubated at 48°C for 20 hours, washed, and underwent a single-base extension step. After that, beadchips were stained, washed, coated, and dried. Finally, signal-intensity data was generated by an Illumina BeadArray Reader. We randomly selected 20% of the total samples and genotyped them in duplicate, and 99.8% concordance was observed. The inconsistent data were excluded from the final analysis.

### Data analysis and functional annotation

The genotyping data for each SNP was analyzed with the BeadStudio software (version 3.3, Illumina, Inc., San Diego, CA, USA). SNPs with poor Illumina design scores were genotyped by sequencing technology on an ABI 3730 DNA analyzer (Applied Biosystems, Inc., Foster City, CA, USA). The protein sequences, structures, homology models, mRNA transcripts, and predicted functions for the SNPs were evaluated by SAMtools.

### Statistical analyses

Hardy-Weinberg equilibrium (HWE) test was performed in both case and control groups with PLINK (version 1.05, http://pngu.mgh.harvard.edu/purcell/plink/)^10^. Significant deviations (*P* < 0.05) from HWE in controls were tested for genotyping quality. The statistical power of the case-control dataset was evaluated using the Genetic Power Calculator software^11^. Difference between the two groups was considered statistically significant when a *P*-value was <0.05 (2-sided). Single-marker analysis was performed by PLINK. Differences in allele and genotype frequencies between the two groups were assessed by Pearson's χ^2^-test or Fisher's exact test. To minimize the number of tests, dominant and recessive models were considered only with those SNPs displaying nominal association under genotypic or allelic models. The strength of association between SNPs was estimated by the odds ratios (OR) with 95% confidence intervals (CI) using unconditional logistic regression, adjusting for age, gender, smoking (yes or no), drinking (yes or no). Bonferroni Correction was used to adjust for multiple comparisons. To test the interaction between individual SNPs, we performed multinomial logistic regression using SPSS software (version 11.5).

## Results

Demographic and clinical characteristics of the overall subjects and patients with primary retroperitoneal liposarcoma enrolled in the study are summarized in Table 1 and Table 2, respectively.

### Genetic balance test

The expected values in the genotype distribution of the genes *MDM2*, *CDK4*, *CDC27*, *FPGS*, *IGFN1*, *and PRAMEF13* were presented in Table 3. All genotype distributions were in HWE, which is a genetic balance test (Table 4).

### Genotyping results

The genotypes and allele frequencies of SNPs in case and control groups are summarized in Table 3.

**Figure 1:**
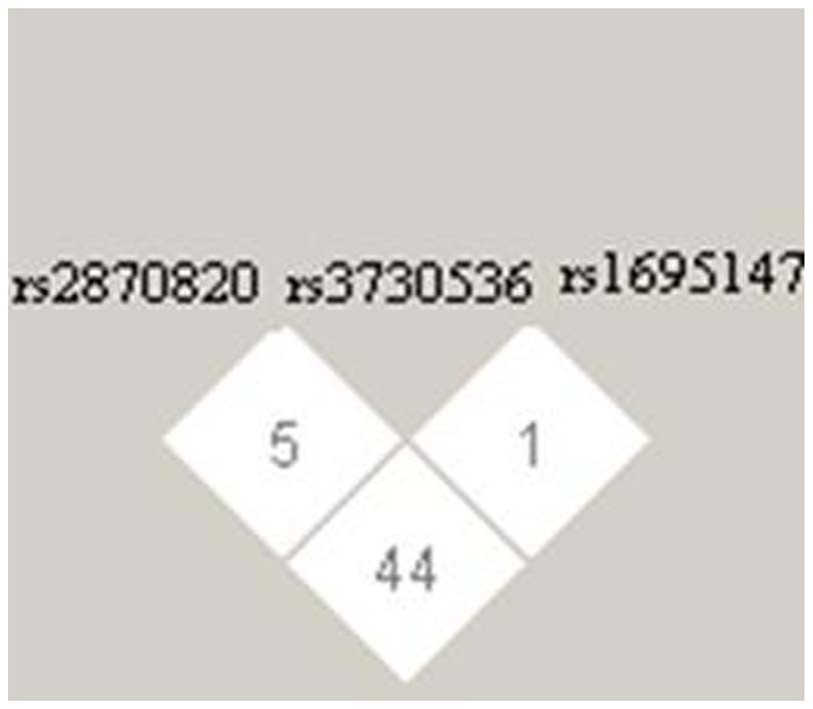
Linkage disequilibrium (LD) of 3 SNPs (rs2870820, rs3730536 and rs1695147).

SNPs of *CDK4* (rs2069502, a tag-SNP), *CDC27* (rs74348171), *IGFN1* (rsrs11803067), and *PRAMEF13* (rs71183793) showed no significant difference between the two groups (*P* > 0.05). Three SNPs (rs2870820, rs1695147, rs3730536) of *MDM2* showed significant differences in single-loci genotypes and allele frequencies between case and control groups (*p* < 0.05). Linkage disequilibrium (LD) of 3 SNPs was analyzed using Haploview (version 4.2), and no haplotype blocks was constructed (Fig. 1). Three SNP are located in intron regions.

**Figure 2:**
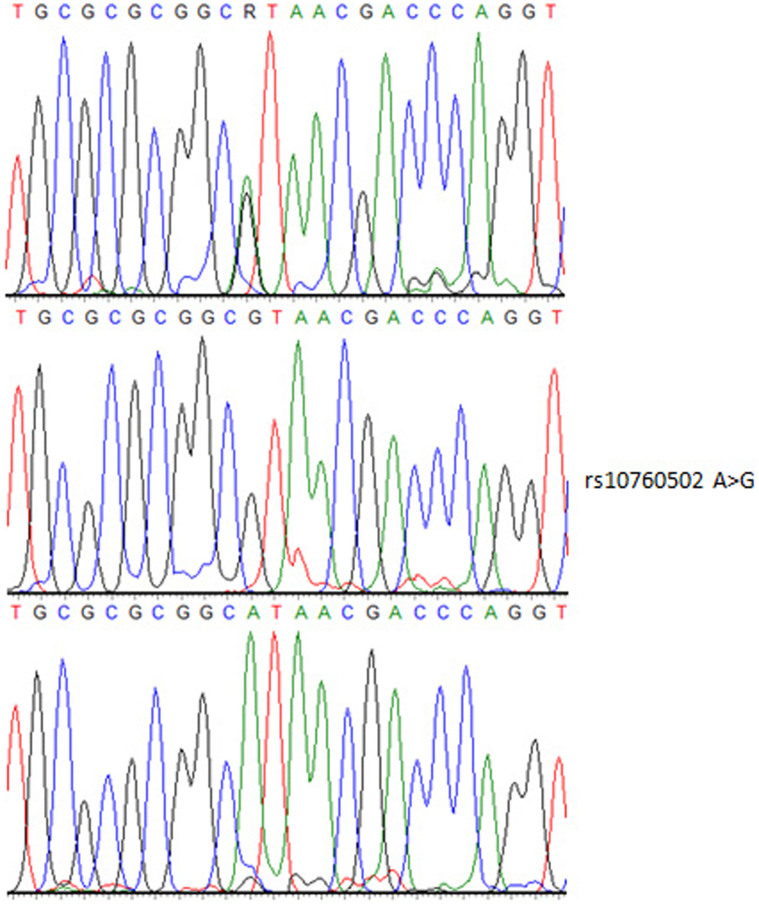
Sanger sequencing to confirm the mutation. Electropherogram showed the heterozygote AG (upper), homozygote mutation GG (middle) and homozygote major allele AA (lower) of rs10760502 located in exon 1 of the FPGS gene.

A SNP of *FPGS* (rs10760502) has shown a significant difference of loci genotype and allele frequencies between case and control [*p* = 0.003, 0.396 (0.240–0.656)]. The case group harbored an A/G genotype more frequently than the control (44% vs.27%; *p* < 0.05) (Table 5). As shown in Figure 1, the genotyping result has been confirmed by sequencing (Fig. 2).

### Protein function prediction

**Figure 3:**
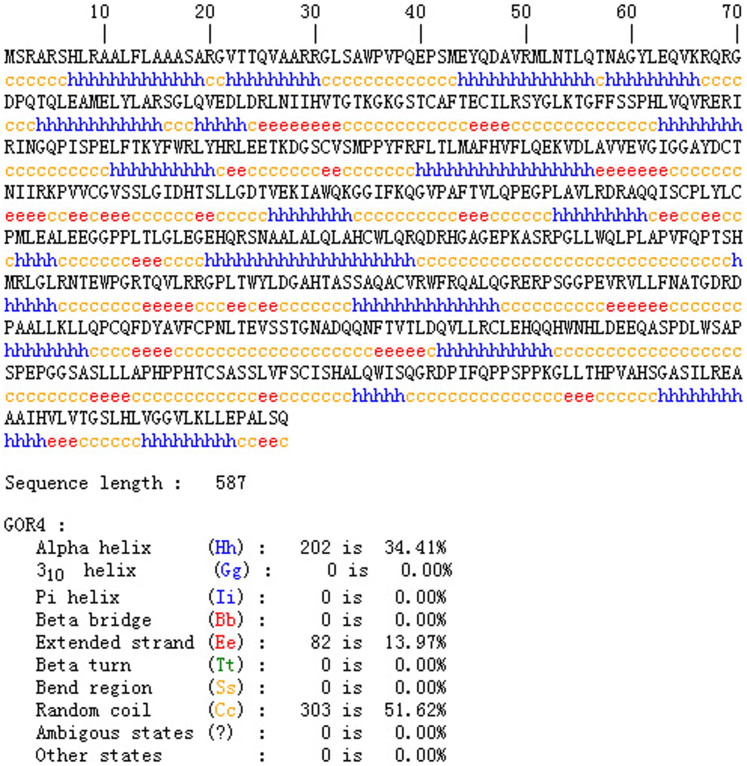
Spatial analysis of two-dimensional structure of proteins using SAMtools software. The native FPGS has 203 α-helix, accounting for 34.58% of the total secondary structure, and 302 random coils, accounting for 51.45% of the secondary structure. The mutated FPGS has 202 α-helix, accounting for 34.41% of the total secondary structure, and 303 random coils accounting for 51.62% of the secondary structure.

**Figure 4:**
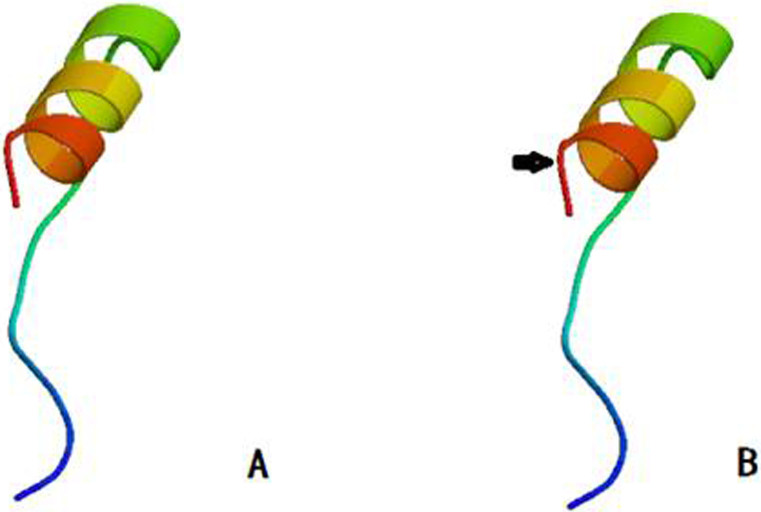
Spatial analysis of three-dimensional structure of proteins. (A) Wild type and (B) Mutant type of FPGS. The arrow represents the change of amino acid in protein translation initiation region.

As shown in Figure 2, SAMtools^12^ (http://samtools.sourceforge.net/) software was used for spatial analysis of two-dimensional structure of proteins. The FPGS^13^,^14^ protein contains 587 amino acids, having a molecular weight of 64609.1 Da. The overall mean hydrophilic coefficient of native FPGS protein is −0.155. The mutated FPGS protein has a molecular weight of 64595.0 Da, with a total average hydrophilic coefficient of −0.156. The native FPGS has 203 α-helix, accounting for 34.58% of the total secondary structure; and 302 random coils, accounting for 51.45% of the secondary structure. The mutated FPGS has 202 α-helix, accounting for 34.41% of the total secondary structure; and 303 random coils, accounting for 51.62% of the secondary structure (Fig. 3). The SWISS-MODEL template library was searched with Blast and HHBlits for evolutionary related structures matching the target sequence in FIG. 3, Protein 3D structure has not changed (Fig. 4).

## Discussion

In the current study, we have found that genetic polymorphism of *FPGS* rs10760502A > G is associated with susceptibility to primary retroperitoneal tumor. The genotype GG, compared to AG or AA, is significantly associated with reduced risk for primary retroperitoneal liposarcomas. Patients carrying rs10760502 GG genotype have lower risk compared to AG or AA, indicating that rs10760502 polymorphism is a potential biomarker for primary retroperitoneal tumor. This mutation is within the first exon of *FPGS*, encoding the 21st amino acid isoleucine. As a missense mutation, ATA → GTA, it replaces isoleucine with valine (I [Ile] → V [Val]). Based on protein structure predication, it has changed the protein 2D structure (conformation), adding an alpha helix and a random coil, without changing functional domains.

We found 3 SNPs located in introns of the *MDM2* gene. Since they did not change the amino acid sequence, there is no evidence that they can affect protein translation process of MDM2. However, we propose that these genetic variants may be in linkage with other functional SNPs or germline mutations within the gene (area), and may serve as tags/markers for hot-spot changes within the genomic region highly associated with the development of primary retroperitoneal tumor. This hypothesis needs to be further investigated. We plan to collect more samples and will examine the association between functional SNPs in MDM2 and other FPGS related genes in a large sample size in the near future.

Two previous studies reported that the allele C of rs1544105 was associated with poor response to methotrexate in patients with rheumatoid arthritis in north India^15^,^16^. Previous research reported that rs1544105 is the independent risk factors of childhood leukemia. This variant was also associated with poor therapeutic outcome for ALL in children^17^. FPGS rs1544105C > T polymorphism may modify FPGS expression and affect treatment outcome in B-cell precursor acute lymphoblastic leukemia (BCP-ALL) patients^17^. Compared to the CT/TT genotypes, the CC genotype was an independent prognostic factor for poor relapse-free survival (RFS) and individuals with the T allele had lower levels of FPGS transcripts. These observations, taken together, provide supporting evidence that FPGS may be involved in tumorigenesis. To the best of our knowledge, our study is the first report that *FPGS* rs10760502 is associated with primary retroperitoneal liposarcoma. Previous studies have demonstrated that downregulation of *FPGS* expression was observed in anti-folate-resistant cell lines^13^,^14^,^18^; however, to date, there is no convincing evidence that primary retroperitoneal liposarcoma is related to abnormal metabolism of folate. To examine the genotype-phenotype association, we are collecting tissue samples to measure the FPGS protein expression profile in primary retroperitoneal tumor using immunohistochemistry (IHC) assays in our ongoing study, which focuses on identification of biomarkers with prognostic and predictive value for those patients.

*FPGS* encodes the folylpolyglutamate synthetase enzyme, which plays a central role in establishing and maintaining both cytosolic and mitochondrial folylpolyglutamate concentrations and is essential for folate homeostasis and the survival of proliferating cells. Dysregulation of *FPGS* directly results in deficiency of folate synthesis. Patients in the absence of folate supplementation have increased risk of DNA breakage, and therefore, may be susceptible to tumorigenesis. There is evidence to support that the lack of folic acid and tumor occurrence has the relevance in leukemia and colon cancer. Folate is required for de novo synthesis of nucleotides A, T, and G (3 of 4 nucleotides required for DNA synthesis). Tumor cells are addicted to folate in order to synthesize DNA prior to cell division.

Antitumor drugs can block tumor cell growth by terminating the folate supply or inhibiting the folate metabolism^19^,^20^,^21^. Folate deficiency results in inappropriate incorporation of uracil into DNA in place of thymine, due to insufficient methylation of dUMP to dTMP. When uracil is removed in the process of DNA repair, transient nicks are formed, leading to chromosome breakage. In addition, A to G mutation occurs more frequently when the folates are deficient, which may eventually promote neoplastic transformation. Another study, however, demonstrated that abundant folates is a double-edged sword, and that timing can dictate whether it is beneficial or harmful^22^. Early folate supplementation may be helpful in the prevention of tumor development; however, after DNA damage and resultant neoplastic transformation have occurred, folate supplementation can accelerate tumor progression.

It has been reported that the incidence of colon cancer is decreasing in US and Canada; however, when folate supplementation was implemented, the incidence of colon cancer showed a transient increase, and then began to decrease^23^. One explanation of this observation is that folate supplementation can increase pre-cancer growth and tumor progression, and therefore, may result in a transient increase in the incidence of colon cancer. Over time, folate supplementation exerts a protective effect, decreases tumor formation, and eventually reduces the incidence of colon cancer.

Our study demonstrated that patients with primary retroperitoneal liposarcomas harbor a mutation in *FPGS*, causing a functional abnormality of folylpolyglutamate synthase, impaired folate synthesis, and resultant DNA breakage. This study suggests that folate supplementation may be used to decrease tumorigenesis and prevent postoperative tumor recurrence.

## Figures and Tables

**Table 1:** Demographic characteristics of the study population

Index	Case (%)	Control (%)	P*
	n = 138	n = 131	
**Age (years)**			0.784
≤40	36 (26.1)	32 (24.5)	
40–60	70 (50.7)	70 (53.4)	
>60	32 (23.1)	29 (22.1)	
**Gender**			0.788
Male	62 (44.9)	61 (46.5)	
Female	76 (55.1)	70 (53.5)	
**Smoking**			0.815
Yes	60 (43.5)	60 (45.8)	
No	78 (56.5)	71 (54.2)	
**Drinking**			0.955
Yes	70 (50.7)	75 (53.2)	
No	68 (49.3)	66 (46.8)	

*P value was from χ^2^ test (2-sided).

**Table 2:** Clinical characteristics of the patients with primary retroperitoneal liposarcoma

Index	No. of patients (%)
**Tumor size (cm)**	
≤5	31 (22.5)
5–10	54 (39.1)
>10	53 (38.4)
**Tumor site**	
Left upper quadrant	18 (13.0)
Right upper quadrant	21 (15.2)
Hypogastrium	44 (31.9)
Pelvic cavity	15 (10.9)
Whole abdomen	40 (29.0)
**Surgical procedures**	
≤1	51 (37.0)
>1	87 (63.0)
**Pathological pattern**	
Highly differentiation	79 (57.2)
Poorly differentiated	40 (29.0)
Myxoid	15 (10.9)
Pleomorphic	4 (2.9)
**Blood transfusion**	
Yes	37 (26.8)
No	101 (73.2)

**Table 3:** SNPs evaluated in this study

				No. of Cases			No. of Controls			Minor Allele Frequency
Gene	SNP	Position	RS No.	M/M	M/m	m/m	M/M	M/m	m/m	Case/Control
MDM2	C > T	g.5356	2870820	106	30	2	85	40	6	0.12/0.2
MDM2	G > T	g.33219	1695147	81	44	13	92	33	6	0.25/0.17
MDM2	A > G	g.15040	3730536	94	37	7	72	46	13	0.18/0.27
CDK4	A > G	g.6500	2069502	110	26	2	107	24	0	0.11/0.09
CD27	A > G	c.1930	74348171	97	35	6	96	30	5	0.17/0.15
FPGS	A > G	g.5114	10760502	64	61	13	89	35	7	0.32/0.19
IGFN1	A > G	c.6071	11803067	81	44	13	72	46	13	0.25/0.27
PRAMEF13	C > T	c.1124	71183793	70	54	14	80	43	8	0.3/0.23

SNP, single-nucleotide polymorphism; RS No., reference SNP identification number; MM, Major/major allele of the SNP; Mm, Major/minor allele of the SNP; mm, minor/minor allele of the SNP.

**Table 4:** Hardy-Weinberg equilibrium genetic balance testing

Genotype		n	aa	ab	bb	a	b	*P*
**rs2870820**	case	78	60	16	2	136	20	0.467
	control	71	46	25	0	117	25	0.0718
**rs1695147**	case	78	46	25	7	117	39	0.199
	control	71	50	18	3	118	24	0.411
**rs3730536**	case	78	53	21	4	127	29	0.329
	control	71	39	25	7	103	39	0.327
**rs2069502**	case	78	62	15	1	139	17	0.931
	control	71	58	13	0	129	13	0.395
**rs74348171**	case	78	55	20	3	130	26	0.497
	control	71	52	19	0	123	19	0.193
**rs10760502**	case	78	44	30	4	110	38	0.593
	control	71	54	17	0	125	17	0.252
**rs11803067**	case	78	46	20	12	117	39	0.199
	control	71	39	25	7	103	39	0.327
**rs71183793**	case	78	40	36	2	116	40	0.0632
	control	71	40	30	1	110	32	0.0765

**Table 5:** Association of genotypes with primary retroperitoneal liposarcomas

Genotype	Case/Control (%)	OR^a^ (95% CI)	*P*^a^
*MDM2* rs2870820			
CC vs. CT/TT	77/65 vs. 23/35	1.082 (1.046–3.103)	0.034
*MDM2* rs1695147			
GG vs. GT/TT	59/70 vs. 41/30	0.584 (0.347–0.982)	0.042
*MDM2* rs3730536			
AA vs. AG/GG	68/55 vs. 32/45	1.762 (1.065–2.916)	0.028
*CDK4* rs2069502			
AA vs. AG/GG	80/82 vs. 20/18	0.876 (0.472–1.626)	0.675
*CDC27* rs74348171			
AA vs. AG/GG	70/73 vs. 30/27	0.884 (0.511–1.530)	0.659
*FPGS* rs10760502			
AA vs. AG/GG	46/68 vs. 54/32	0.396 (0.24–0.656)	<0.001
*IGFN1* rs11803067			
AA vs. AG/GG	59/55 vs. 41/45	1.171 (0.715–1.917)	0.532
*PRAMEF13* rs71183793			
CC vs. TT/CT	77/65 vs. 23/35	0.642 (0.391–1.056)	0.081

^a^OR (95% CI) and *P* value were calculated from logistic regression model adjusted for age, gender, smoking and drinking.
